# Neurological pathogenesis of SARS-CoV-2 (COVID-19): from virological features to clinical symptoms

**DOI:** 10.1186/s41232-021-00165-8

**Published:** 2021-05-07

**Authors:** Yoshitaka Kase, Hideyuki Okano

**Affiliations:** 1 Department of Physiology, Keio University School of Medicine, 35 Shinanomachi, Shinjuku-ku, Tokyo, 160-8582 Japan; 2 Department of Geriatric Medicine, Graduate School of Medicine, The University of Tokyo, Bunkyo-ku, Tokyo, 113-8655 Japan

**Keywords:** Coronavirus (CoV), COVID-19, Neural disorder, SARS-CoV-2

## Abstract

Since the worldwide outbreak of coronavirus disease 2019 (COVID-19) in 2020, various research reports and case reports have been published. It has been found that COVID-19 causes not only respiratory disorders but also thrombosis and gastrointestinal disorders, central nervous system (CNS) disorders, and peripheral neuropathy. Compared to other disorders, there are low number of research reports and low number of summaries on COVID-19-related neural disorders. Therefore, focusing on neural disorders, we outline both basic research and clinical manifestations of COVID-19-related neural disorders.

## Introduction

As coronavirus disease 2019 (COVID-19) has spread around the world [1] and numerous reports have been published; it has become clear how SARS-CoV-2 can infect humans and exert pathogenicity [2].

It has also been found that COVID-19 causes not only lung and vascular damage but also neural damage, and in this review, we first describe the general virological features of not only SARS-CoV-2 but also other coronaviruses that infect humans. Next, we summarize the neurotropism of six viruses other than SARS-CoV-2 that are known to infect humans. Then, we outline the virological characteristics of SARS-CoV-2 and consider its neurotropism. Finally, we review the clinical reports so far to summarize the most common symptoms observed in COVID-19-related neural disorders.

## Coronavirus genomic structure and characteristics

First, we outline the characteristics that various coronaviruses (CoVs) have in common.

**Fig. 1:**
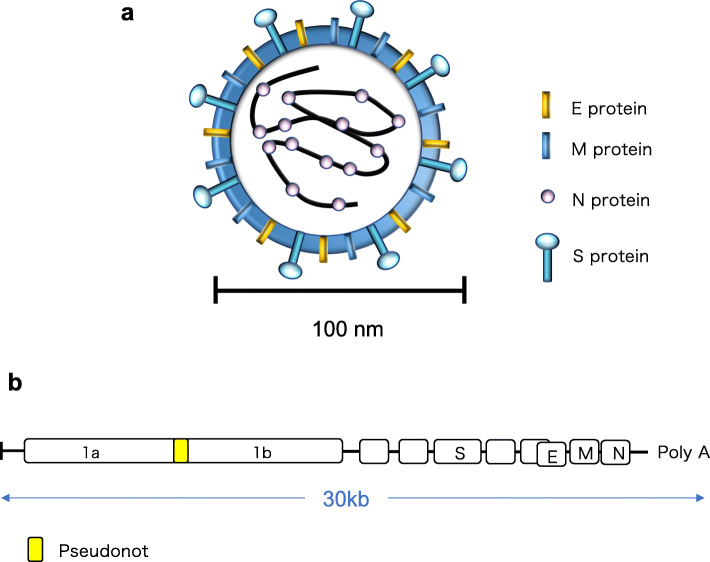
Schematic diagram of the coronavirus structure and genomic structure. **a** The coronavirus genome is single-stranded RNA, which together with nucleocapsid (N) protein constitutes the nucleocapsid. There is an envelope that encloses the nucleocapsid, which consists of spike (S) protein, integral membrane (M) protein, and envelope (E) protein. **b** ORF1a and ORF1b continue to exist from the 5' end to approximately 20 kbp. Since the pseudoknot structure exists between the two, two patterns of translation products of the 1a region or 1a + 1b region are generated. Other ORFs encode S protein, E protein, M protein, N protein, etc

CoV particles are spherical with a diameter of approximately 100 nm and have an RNA genome [3, 4]. The nucleocapsid (N) protein binds to genomic RNA to form the nucleocapsid, and the spike (S) protein, integral membrane (M) protein, and envelope (E) protein form an envelope that encloses the nucleocapsid [3] (Fig. 1a).

There are multiple ORFs in the genomic RNA of CoVs, and ORF1a and ORF1b are characteristic. Between ORF1a and ORF1b, there is a structure called a pseudoknot in which ORF1a and ORF1b overlap, and this structure causes a frame shift with a certain probability when the ribosome proceeds with RNA translation [3–5]. That is, CoV genomic RNA is translated into two types of proteins, the ORF1a region or the ORF1a + ORF1b region. The ratio between the two is higher in the ORF1a region than in the ORF1a + ORF1b region. Proteolytic enzymes that degrade themselves are mainly translated from the ORF1a region, and RNA-dependent RNA polymerase and helicase are produced by cleaving the translation products from the ORF1b region [3, 6] (Fig. 1b).

## Processes involved in progression from the establishment of coronavirus infection to replication and release

The processes involved in progression from infection to the release of virus particles of coronavirus can be divided as follows: (1) adsorption, (2) invasion, (3) production of RNA polymerase, (4) genome replication, (5) translation, (6) assembly, and (7) release [3] (as will be described later, the receptors used for adsorbing coronavirus differ depending on the type of virus).

1. Adsorption: the S protein binds to receptors on the host cell surface.
2. Invasion: viral particles invade the cell, and the viral genome is released into the cytoplasm.
3. Production of RNA polymerase: since the genome of coronavirus is a (+) RNA strand that can function as mRNA, RNA polymerase is translated.
4. Genome replication: a (−) strand RNA is synthesized using genomic RNA as a template, and then genomic RNA is synthesized using it as a template.
5. Translation: viral constituent proteins are produced using the produced (−) strand RNA as a template.
6. Assembly: the N protein binds to genomic RNA to form the nucleocapsid. S protein, E protein, and M protein are combined.
7. Release: a new virus is released in the form of an envelope derived from the endoplasmic reticulum membrane of the host cell.

## Characteristics of neural disorders caused by coronaviruses other than SARS-CoV-2 that infect humans

Here, we describe the effects of various coronaviruses other than SARS-CoV-2 that infect humans [7, 8] on neural cells (Table 1).

**Table 1:**
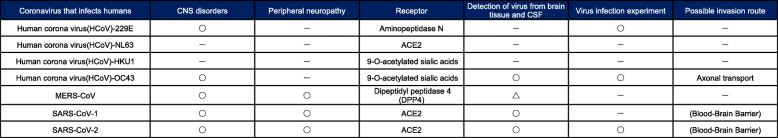
Summary of neural disorders of coronaviruses that infect humans, including SARS-CoV-2

The CoVs that have been reported to infect humans include seven types of viruses belonging to the α-coronaviruses or β-coronaviruses among Orthocoronaviruses. Human coronavirus (HCoV)-229E [9] and HCoV-NL63 [10] belong to the α-coronaviruses, and HCoV-HKU1 [11], HCoV-OC43 [12], Middle East respiratory syndrome corona virus (MERS)-CoV [13], severe acute respiratory syndrome coronavirus (SARS-CoV)-1 [14], and SARS-CoV-2 belong to the β-coronaviruses.

HCoV-229E, HCoV-NL63, HCoV-HKU1, and HCoV-OC43 are the causative viruses of the so-called winter cold. There are fewer studies on neural disorders caused by these viruses than by SARS-CoV-1 or SARS-CoV-2. This is because, when the existence of these viruses was confirmed, they had already become established as the causative virus of the winter cold in human beings, and they were not paid much attention as research subjects.

Nevertheless, among these four viruses, HCoV-229E [15] and HCoV-OC43 have been reported to cause neural disorders. HCoV-229E and HCoV-OC43 use aminopeptidase N [16] and 9-O-acetylated sialic acids [17] as receptors for adsorption. It has been reported that HCoV-OC43 may cause multiple sclerosis [15] and encephalitis [18, 19], and experiments have been conducted to infect neural cells in vitro [20]. In addition, axonal transport is cited as a possible route of infection of the nervous system for HCoV-OC43 [21].

MERS-CoV is a dromedary-hosted virus that was identified in Saudi Arabia in 2012. Even now, there are sporadic cases of MERS. MERS is a very serious and fatal disease with a case fatality rate of 35% [13, 22]. However, it has also been reported that 0.15% of Saudi Arabians have anti-MERS-CoV antibodies [23]; taking these potentially infected people into account, the case fatality rate can be estimated to be approximately 2%.

Dipeptidyl peptidase-4 (DPP4) has been identified as a receptor for MERS-CoV infection [24]; however, few reports have verified whether DPP4 is expressed in neural cells. Although neural disorders due to MERS-CoV infection have been reported [25, 26], the low number of reports may be because DPP4 expression is not detectable in the nervous system. Thus, MARS-related neural disorders may be limited to those caused by systemic inflammation and angiopathy.

SARS-CoV-1 was identified in 2003 and causes SARS [14], which is a serious disease with a case fatality rate of up to 10–20%. There is a case report that SARS causes neural disorders [27], and there is also a report that SARS-CoV-1 caused neural cell death in an experiment using mice [28]. The receptor for SARS-CoV-1 is angiotensin-converting enzyme 2 (ACE2), which is the same as that for SARS-CoV-2 [29]. As described later, there are some reports showing that ACE2 is expressed in neural cells, and SARS-CoV-1 may thus directly infect neural cells and cause neural disorders.

## Characteristics of SARS-CoV-2 infection

Here, we describe SARS-CoV-2 in detail in terms of its genomic structure, similarity with other coronaviruses, and differences.

When SARS-CoV-2 is adsorbed on the host cell, ACE2 acts as a receptor, similar to SARS-CoV-1 [29]. Adsorption is initiated by the binding of the S protein and ACE2. The virus that has the gene with the highest sequence similarity to an S protein is RaTG13, a type of bat-hosted bat coronavirus (Bat-CoV). Wrobel et al. compared S protein of SARS-CoV-2 with one of RaTG13 and found that while they are structurally similar to each other, SARS-CoV-2 S protein is more stable. They also reported that the SARS-CoV-2 S protein can bind to ACE2 approximately 1000 times more strongly than the RaTG13 S protein [30].

Furthermore, focusing on the receptor-binding domain (RBD) in the sequence encoding an S protein, it shares a characteristic sequence with SARS-CoV-2 and Pangolin-CoV. In the RBD within the S1 sequence of SARS-CoV-2, six amino acids play a decisive role in the binding of ACE2 receptors, and the coding regions of these six amino acids are shared between SARS-CoV-2 and Pangolin-CoV [29].

**Fig. 2:**
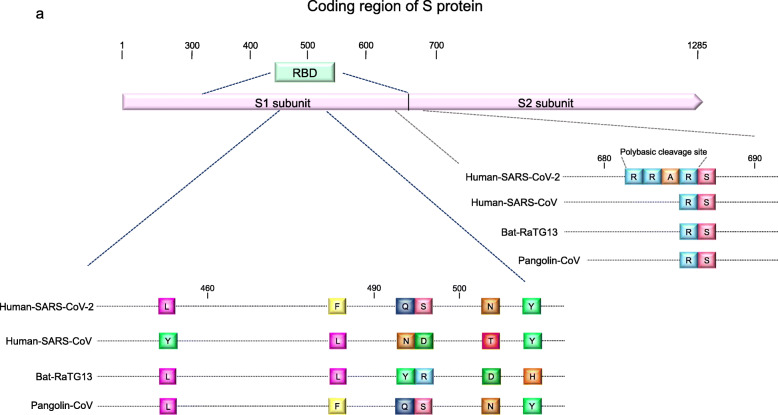
Consideration of the origin of SARS-CoV-2; common sequence of various coronaviruses and SARS-CoV-2. **a** The RBD within the S1 subunit has a common amino acid sequence between SARS-CoV-2, RATG13, and Pangolin-CoV. The cleavage site has an amino acid sequence (RRAR) unique to SARS-CoV-2. (Modified from Andersen et al. [31])

It is also known that the serine protease transmembrane protease serine 2 (TMPRSS2) cleaves the spikes of SARS-CoV-2 and increases the infectivity of SARS-CoV-2 [29]. TMPRSS2 is a protease that SARS-CoV-1 also uses to cleave spikes. SARS-CoV-2 has a cleavage sequence (amino acid sequence: RRAR) present in the S1 and S2 subunits of the S protein, which is cleaved by TMPRSS2. This sequence is unique to SARS-CoV-2 and is not found in SARS-CoV-1, Pangolin-CoV, or Bat-CoV (RaTG13) [31] (Fig. 2).

These main characteristics of SARS-CoV-2 would be the basis for the establishment of SARS-CoV-2-specific infection. Based on these structural characteristics of the viral genome, it is likely that SARS-CoV-2 was not generated simply by mutation of SARS-CoV-1 or MERS-CoV but rather by recombination with RaTG13 or Pangolin-CoV in some intermediate host animals.

## SARS-CoV-2 infection in neural cells

### Is it possible to adsorb to neural cells?

SRAS-CoV-2 establishes its infection of cells by the S protein/ACE2-mediated mechanism as described above. However, does SARS-CoV-2 infection occur in the nervous system? To address this point, there are several reports in which human-induced pluripotent stem cells (hiPSCs) were used to generate neurons and brain organoids to investigate whether ACE2 is expressed in neural cells [32–37]. Through a database search [38], although the expression level of ACE2 was low, its expression was confirmed in immature neurons derived from embryonic stem cells (ESCs). On the other hand, while the expression level of ACE2 is low in the entire brain (Allen Brain Atlas: Human brain (https://human.bainmap.org/microarray/search)), its expression level is high in some parts of the brain, including the striatum (i.e., the locus of necrotizing encephalopathy associated with COVID-19 [39]) and the choroid plexus, which is relevant to the case report on the detection of SARS-CoV-2 in cerebrospinal fluid (CSF) [40].

Several studies have been reported in which pseudovirus or SARS-CoV-2 was added to brain organoids prepared from iPSCs to examine whether infection was established. When SARS-CoV-2 is added to neural progenitor cells and brain organoids prepared from human iPSCs in vitro, infection is established, and viral proliferation and neuronal cell death are induced. In this system, antibodies against ACE2 or CSF (containing IgG antibodies specific to S protein) from COVID-19 patients prevent neural infection with SARS-CoV-2 [32], indicating that ACE2 acts as a receptor for SARS-CoV-2 in neural cells.

Ramani et al. performed an experiment in which SARS-CoV-2 was infected with brain organoids prepared from hiPSCs. SARS-CoV-2 is more likely to infect neurons than neural progenitor cells (NPCs), causing changes in tau distribution, hyperphosphorylation of tau in infected neurons, and neuronal death [35]. Experiments by Yi et al. also showed that SARS-CoV-2 pseudovirus is more likely to infect brain organoid neurons than NPCs [36]. Pellegrin et al. also generated brain organoids containing the choroid plexus from hiPSCs and conducted an infection experiment with SARS-CoV-2 spike pseudovirus and live virus. They demonstrated that choroid plexus epithelial cells were infected with the virus and suggested that those cells were damaged [41].

Furthermore, not all neuronal subtypes have the same susceptibility, and dopaminergic neurons are particularly susceptible to SARS-CoV-2 infection [34]. According to the database of Hodge et al. (https://celltypes.brain-map.org) [42], ACE2 expression does not appear in any neuronal subtype according to the single-nucleus RNA-sequencing profile of the middle temporal gyrus of the human cortex. From these facts, it is possible that the expression of ACE2 differs among brain regions in the adult human brain and among neuronal subtypes. Alternatively, it is possible that receptors for SARS-CoV-2 other than ACE2 exist in the nervous system, and they may play an important role in the neural infection of SARS-CoV-2.

### What is the infection route by which SARS-CoV-2 reaches the brain?

The first possible route for SARS-CoV-2 to enter the brain is across the blood-brain barrier (BBB). There are several research reports on whether the virus can cross the BBB, with reports showing that subunit S1 of the SARS-CoV-2 S protein reached the brain across the mouse BBB [43]. In this report, the virus administered intranasally also reached the brain, but it was approximately one-tenth of that observed after intravenous administration. It should also be noted that as a result of systemic inflammation caused by COVID-19, the BBB is disrupted [44] and becomes a viral invasion route.

In addition to these reports, there is a study in humans that proposed a route of viral entry from the olfactory mucosa into the brain rather than the BBB. Meinhardt et al. investigated the nasopharynx and brain, which are likely to be the first sites of viral infection and viral replication in 33 patients (22 males and 11 females) who died of COVID-19. They found the presence of S protein in cells within the olfactory mucosal layer. In addition, in the olfactory mucosal layer, endothelial tissue and nerve tissue are in close proximity, and they report that the virus may use this to invade the brain; SARS-CoV-2 S protein has been detected in neurons in patients with COVID-19 [45].

There is also a review article advocating the possibility of transsynaptic transmission of SARS-CoV-2 from the peripheral nerve, which still needs to be elucidated more unequivocally [46].

Although whether SARS-CoV-2 can be detected in the brain or cerebrospinal fluid in pathological autopsy is still under debate, in some reports, SARS-CoV-2 S protein was detected in the patient’s pathological specimens, and a correlation between clinical symptoms and histological pathological features was observed [32].

## COVID-19-related disorders: clinical findings

Finally, we describe the clinical features of neural disorders caused by COVID-19. Many neural disorders and diseases related to COVID-19 have been reported, and there are reports summarizing the cases. Prior to February 2020, there were a series of reports of ischemia associated with lung damage and thrombosis/embolism, and since then, reports reminiscent of the direct action of the virus on neurons have been reported [47].

Mao et al. [48] demonstrated the following clinical features among 214 total patients infected with SARS-CoV-2: 78 patients (36.4%) showed symptoms of neural disorders, including CNS disorders (53 [24.8%]) and peripheral neuropathy (19 [8.9%]). The most common symptoms reported in patients with CNS symptoms were dizziness (36 [16.8%]) and headache (28 [13.1%]).

Helms et al. [49] reported an observational study of neural disorders in 58 of 64 COVID-19 patients. Interestingly, all 13 patients who underwent the head MRI had cerebral perfusion disorders.

Romero-Sánchez et al. [50] reported that of 841 patients admitted with COVID-19, 57.4% developed some form of neurological symptoms. Headache (14.1%) and dizziness (6.1%) were the most common neural disorders  with mild symptoms, and severe disorders such as consciousness disorder (19.6%) were confirmed in elderly patients and severe cases. Peripheral neuropathy (3.1%), autonomic imbalance (2.5%), and Guillain-Barré syndrome (*n* = 1) were also reported, but less frequently.

Additionally, in a report released in 2021, in 33 patients who died of COVID-19, COVID-19-related neurological changes included disturbance of consciousness (*n* = 5), intraventricular hemorrhage (*n* = 2), headache (*n* = 2), and behavioral changes (*n* = 2) [45].

We extracted some summaries of case reports and found that mild symptoms such as headache and dizziness were observed rather than severe symptoms. However, since these headaches and dizziness are symptoms induced by inflammation associated with other infectious diseases and stress, it will be necessary to follow-up on whether they are truly symptoms associated with COVID-19.

## Sequelae of COVID-19

Centers for Disease Control and Prevention (CDC) reports on COVID-19 sequelae; various symptoms that continue beyond the acute phase of COVID-19 are defined as long COVID [51]. Among them, those related to neural disorders are difficulty thinking or concentrating (brain fog), depression or anxiety, headache, loss of smell or taste, and dizziness on standing. Among them, brain fog is attracting attention and is a long-term symptom of poor concentration. When this was first reported, it was a symptom that appeared even after the inflammation in the acute phase had healed, and the cause was unknown. However, as mentioned above, since it has been reported that SARS-CoV-2 infects the central nervous system and causes neural cell deaths, brain fog may be a peculiar long-term symptom of COVID-19.

In addition, dysgeusia and olfactory dysfunction are often reported as sequelae. Most patients with these disorders recover in 14 days, but some patients remain symptomatic for longer periods [52]. ACE2 may be expressed in the taste buds [53], and these long-lasting disorders may be directly affected by the virus.

## Conclusion

In summarizing the neural disorders caused by SARS-CoV-2, we described the characteristics of coronavirus, the virological characteristics of SARS-CoV-2, and the clinical characteristics of neural disorders. The expression of ACE2, the receptor used for adsorption during SARS-CoV-2 infection, is observed in neural cells. However, the expression level and the region where it is expressed in the brain seem to be limited. Based on various reports, there may be neural disorders caused by the direct action of the virus, in addition to systemic inflammation and thrombi/emboli. The neurological symptom associated with COVID-19 is often headache or dizziness, but neural complications seem to worsen in moderate and severe cases.

Furthermore, it can be expected that the pathophysiology of COVID-19-related neural disorders will change as the virus mutates in the future. Infectious diseases such as coronavirus, which generally have many asymptomatic patients, are dominated by mutant viruses that easily spread. Since the possibility of increased neurotoxicity cannot be ruled out depending on the mutation, continuous efforts in basic and clinical research are required.

## Abbreviations

ACE2: Angiotensin-converting enzyme 2
BBB: Blood-brain barrier
CDC: Centers for Disease Control and Prevention
CSF: Cerebrospinal fluid
CNS: Central nervous system
CoV: Coronavirus
COVID-19: Coronavirus disease 2019
ESC: Embryonic stem cell
E protein: Envelope protein
hiPSC: Human-induced pluripotent stem cell
HCoV: Human coronavirus
MERS-CoV: Middle East respiratory syndrome coronavirus
M protein: Membrane protein
N protein: Nucleocapsid protein
RBD: Receptor binding domain
SARS-CoV-2: Severe acute respiratory syndrome coronavirus 2
S protein: Spike protein
TMPRSS2: Transmembrane protease serine 2
